# Evaluation of presepsin as a diagnostic tool in newborns with risk of early-onset neonatal sepsis

**DOI:** 10.3389/fped.2022.1019825

**Published:** 2023-01-09

**Authors:** Iva Pospisilova, Helena L. Brodska, Marketa Bloomfield, Klara Borecka, Jan Janota

**Affiliations:** ^1^ Department of Clinical Chemistry, First Faculty of Medicine, Thomayer University Hospital and Charles University, Prague, Czech Republic; ^2^ Department of Pediatrics, First Faculty of Medicine, Thomayer University Hospital and Charles University, Prague, Czech Republic; ^3^ The Institute of Medical Biochemistry and Laboratory Diagnostics, First Faculty of Medicine, General University Hospital and Charles University, Prague, Czech Republic; ^4^ Department of Immunology, Second Faculty of Medicine, Motol University Hospital and Charles University, Prague, Czech Republic; ^5^ Department of Obstetrics and Gynecology, Neonatal unit, Second Faculty of Medicine, Motol University Hospital and Charles University, Prague, Czech Republic; ^6^ Institute of Pathological Physiology, First Faculty of Medicine, Charles University, Prague, Czech Republic; ^7^ Department of Neonatology, First Faculty of Medicine, Thomayer University Hospital and Charles University, Prague, Czech Republic

**Keywords:** biomarker, inflammation, neonatal sepsis, newborns, presepsin

## Abstract

**Objectives:**

To evaluate the efficacy of presepsin (P-SEP) as a potential biomarker of early-onset neonatal sepsis (EOS) and compare it to other routinely used markers of inflammation. To establish the cut-off values of P-SEP for EOS.

**Study design:**

184 newborns were prospectively recruited between January 2018 to December 2020. Newborns >34th gestational week with suspected infection were included up to 72 h after delivery, and divided into three categories (i.e., unlikely, possible, and probable infection) based on risk factors, clinical symptoms and laboratory results. Values of plasma P-SEP were sequentially analyzed.

**Results:**

Median values of P-SEP in newborns with probable infection were significantly higher compared to healthy newborns (*p* = 0.0000013) and unlikely infection group (*p* = 0.0000025). The AUC for discriminating the probable infection group from the unlikely infection group was 0.845 (95% Cl: 0.708–0.921). The diagnostic efficacy of P-SEP was highest when used in combination with IL-6 and CRP (0.97; 95% CI: 0.911–0.990). The optimal cut-off value of P-SEP was determined to be 695 ng/L.

**Conclusion:**

P-SEP, when combined with IL-6 and CRP, may be utilized as a negative predictive marker of EOS (NPV 97.2%, 95% CI: 93.3–101), especially in newborns at low to medium risk of infection.

## Introduction

Inflammation is an organism's defensive response to damage to its integrity. The hallmark of innate immunity is the ability to recognize and react to a broad spectrum of pathogens mediated by pathogen-associated molecular patterns (1). An excessive, underregulated systemic inflammatory response to infectious stimuli underlies the development of sepsis (2).

Despite the decreasing incidence of early-onset neonatal sepsis (EOS) in the last 20 years, mainly owing to the EOS risk factors awareness, the implementation of group B *Streptococcus* (GBS) screening programs for expectant mothers and antibiotic prophylaxis during delivery, sepsis remains one of the most common causes of newborn mortality and morbidity (3). Therefore, timely diagnosis and early initiation of appropriate treatment is of utmost importance.

The diagnosis of neonatal sepsis is challenging and, to this day, neither a single over-arching definition of neonatal sepsis nor any unified diagnostic criteria exist (4). The established consensual definition put forth by the International Pediatric Sepsis Consensus Conference in 2005 defined pediatric sepsis as the presence of >2 criteria for Systematic Inflammatory Response Syndrome (SIRS) (one of which must be temperature or leukocyte count) in the setting of suspected or proven infection. However, such diagnostic criteria are suboptimal in term and premature newborn infants (5).

Aiming to increase the prognostic accuracy, a new definition of sepsis was accepted in 2016 (6). This definition moves away from nonspecific risk factors towards the emphasis on organ dysfunction quantified by a scoring system—the sequential organ failure assessment (SOFA score) or its abbreviated version (qSOFA). However, the applicability of this definition extends poorly beyond the adult population it was designed and tested for. Most importantly, it does not appropriately reflect the pediatric variations such as age-specific vital signs parameters (e.g., blood pressure, heart rate, breathing frequency, cognitive state) (7).

For these reasons, positive blood culture remains the gold standard for defining newborn sepsis in spite of its drawbacks. Blood culture process is a time-consuming method, requires a larger sample volume (8), its sensitivity is low and the results are often compromised by prenatal antibiotic use (9–11).

Due to the need to distinguish between neonatal systemic infectious response (overt sepsis) and non-infectious SIRS, new biomarkers indicating/excluding the presence of an infectious agent are highly sought-after to help guide the therapeutic decisions, especially when blood culture results are negative.

All currently available biomarkers of infection have relatively low specificity and sensitivity affected by postnatal physiology, delayed dynamics and individually variable metabolism, and disparities in reference ranges, all of which are highly dependent on the gestational age of the child.

The complete blood count with differential (CBC) is used in the diagnosis of neonatal sepsis; however, a single time-point sampling has a low predictive value. C-reactive protein (CRP) has only limited diagnostic potential in the early stages of sepsis due to the delayed induction of its hepatic synthesis and the presence of other infection-independent inductive factors. In addition, CRP levels on admission are similar in neonates with positive and negative blood cultures for suspected EOS (12). The utility of procalcitonin (PCT), one the most widely used markers of sepsis, is hampered in neonates mainly by its dependence on gestational and postnatal age, as the levels of PCT can be physiologically elevated up to 48 h after the delivery (13, 14). Lastly, the use of interleukin 6 (IL-6), a highly sensitive predictor of EOS, is limited by its low specificity due to its responsiveness to a myriad of other non-infectious conditions (3). Although the diagnostic efficacy of the aforementioned markers is improved when combined, an identification of a singular marker of EOS with a high degree of sensitivity and positive predictive value (PPV) would benefit the clinical practice.

Presepsin (P-SEP), the soluble N terminal fragment of CD14, is a novel promising biomarker with higher prognostic potential than PCT in early stages of sepsis. It is physiologically expressed on the surface of monocytes and macrophages and is excessively shed into the systemic circulation upon stimulation by exogenous antigens of bacterial origin, such as bacterial lipopolysaccharide. The concentration of P-SEP in blood starts to increase within 2 h after induction, peaks at 3 h and remains elevated for up to 4–5 h (15). As such, P-SEP induction precedes the other routinely used markers of inflammation. To practical advantage, the analysis may be performed *via* point-of-care testing and requires minimal amount of whole blood or plasma (120 µl).

The focus of this study is to evaluate the utility of P-SEP in the diagnosis of EOS and to compare its performance with markers of inflammation that are currently in routine clinical use.

## Materials & methods

Newborns born between 34th and 42nd week of gestation, who were admitted to the neonatal intensive care (NICU) of Thomayer University Hospital in Prague, Czech Republic, from January, 2018 to December, 2020 with clinical signs of infection developing within the first 72 postnatal hours were selected for this prospective study. Sequential sampling was performed at 12–24 h (T1) and 36–72 h (T2) intervals from the initial sampling (T0). Subjects selected for this study were sampled for an extra micro sample or „cap” (0.5 ml) during routine blood collections. Routinely used markers of inflammation (IL-6, PCT, CRP) were measured on the day of sampling from fresh serum, the CBC was measured from samples collected in EDTA-coated test tubes. For P-SEP concentration, plasma was centrifuged, aliquoted and stored frozen at −20 °C temperature to enable serial analysis.

Study subjects were divided into groups based on published criteria (16), as follows:

I. **Risk factors (A)**
  a. mother group B *Streptococcus* positive
  b. clinical &/or histological signs of maternal chorioamnionitis
  c. premature rupture membranes ≥18 h
  d. gestational age less than 37th week of pregnancy
II. **Clinical symptoms (B)**
  a. signs of respiratory distress or apnea
  b. tachycardia/bradycardia
  c. arterial hypotension
  d. hypothermia/hyperthermia
  e. lethargy/increased irritability
  f. vomiting/fluid intolerance, signs of ileus
III. **Laboratory findings (C)**
  a. white blood cell count below 5 × 10^9^/L
  b. CRP > 10 mg/L

**Figure 1:**
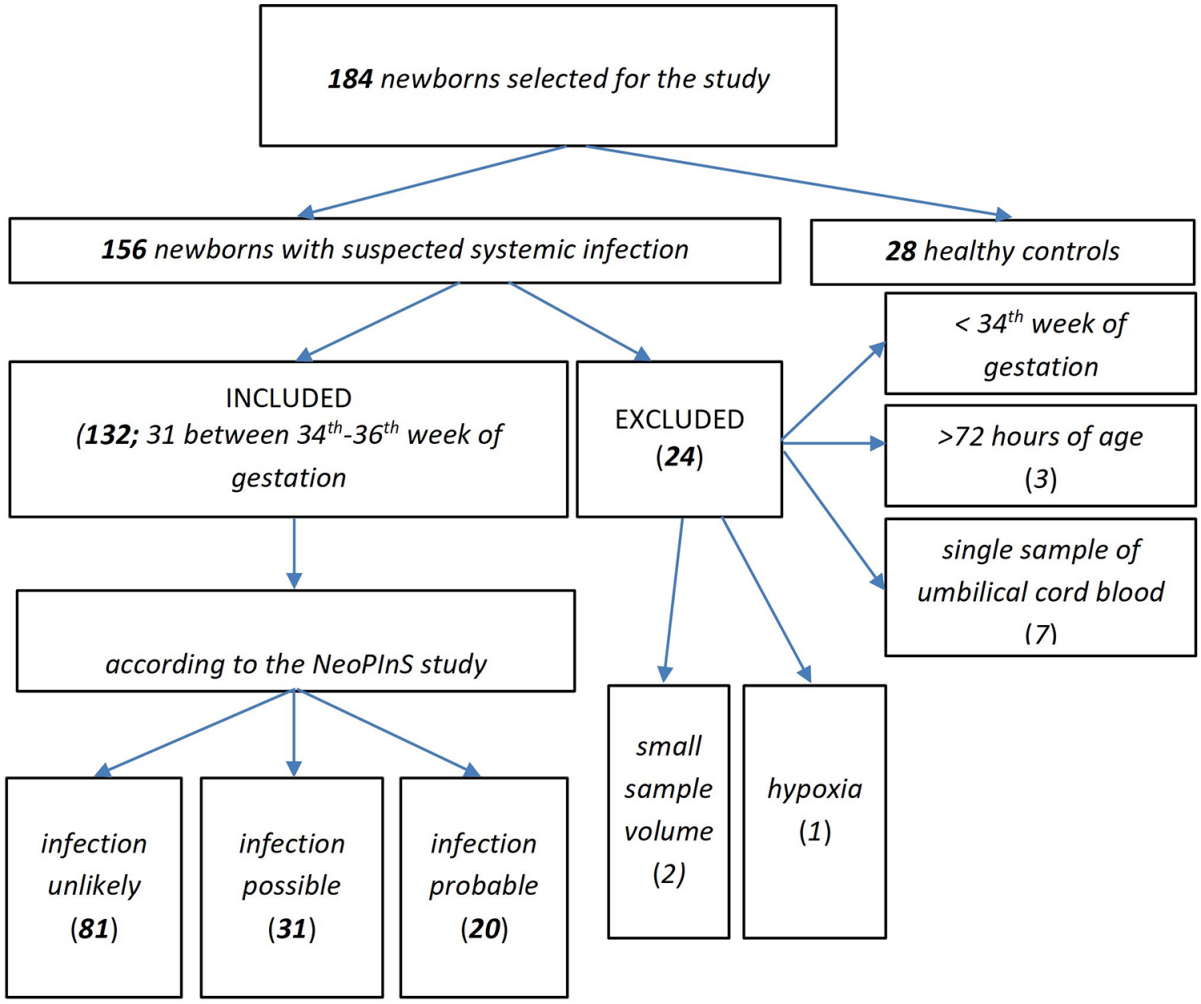
Representation of newborns in the study.

The selection of at least 1 criterion from each of the subgroups would earn 1 point, for a total of 3 points. Newborns were divided into the following groups (see Figure 1, Table 1) based on the obtained score from the subgroups A, B & C in addition to the blood culture (BC) results:

• *Unlikely* infection (low-risk, R1)—BC negative + total score 0–1 point
• *Possible* infection (medium-risk, R2)—BC negative + total score 2 points
• *Probable* infection (high-risk, R3)—BC negative + total score 3 points
• *Proven* infection—BC positive + total score ≥ 1 point

**Table 1.** The clinical criteria of neonatal sepsis. GBS-group B *Streptococcus*, PROM-premature rupture of membranes, RDS-respiratory distress syndrome.

	Groups of newborns			
	controls	low risk	medium risk	high risk
total score (points)	0	1	2	3
symptom	no finding	risk factors* gestational age <37 weeks or GBS positive or PROM > 18 h or signs of maternal chorioamnionitis	risk factors* + *CRP > 10 mg/L* or clinical symptoms* (RDS/apnoe or tachycardia/bradycardia or arterial hypotension or hypothermia/hyperthermia or floppy infant, irritability/lethargy or vomiting/ileus)	risk factors* *+* clinical symptoms* *+* *CRP > 10 mg/L*

* One point was given if one or more of risk factors/clinical symptoms were positive.

The control group (0 points; *n* = 28) was recruited from healthy newborns with physiologic postnatal period, sampled up to 9 h after delivery. Umbilical cord blood samples were obtained from the majority of the healthy subjects, venous blood was sampled in the minority. Plasma levels of P-SEP and CRP were measured.

The study was carried out according to the Declaration of Helsinki, with the consent of the local ethics committee (Docket No.: G-17–06-06). Written informed consents to participate in the study were obtained from legal guardians of all included subjects.

## Analytic testing & methodology

Serum concentrations of PCT and IL-6 were measured using the electrochemiluminescent (ECLIA) assays in the Cobas (Roche Diagnostics) analyzer. CRP was measured using immuno-turbidometry in the Cobas/Modular (Roche Diagnostics) analyzer. P-SEP plasma concentration was measured using the PATHFAST™ analytic system (Mitsubishi, MEDESA s.r.o.) based on chemiluminescent enzymatic immunoanalysis (CLEIA). Blood count and differential was measured *via* fluorescent flow cytometry using the XN 3000 analyzer (Sysmex).

### Microbiologic testing

0.5–1 ml of blood was added to cultivation media (Bactec Peds Plus/F) and incubated at a temperature of 37 °C for 7 days. Positive bottles were subcultured on blood agar and MacConkey agar. Isolated microbes were indentified using standard bacteriological methods.

### Statistical analysis

Receiver operating characteristics (ROC) were used for the discrimination between the risk of infection (low, medium, high) based on the basal levels of presepsin and further markers. The relationship between the basal presepsin levels and risk of infection was further investigated by robust Kruskal-Wallis test followed Dunn's multiple comparisons with Bonferroni correction. The relationships between the time profiles of presepsin levels and degrees of infection was evaluated using the ANOVA model consisting of subject factor, between-subject factor Risk of infection (low, medium, high), within-subject factor Hour (0, 12–36, 48–72) and Risk of infection × Hour interaction followed by Bonferroni multiple comparisons. The original continuous data were transformed by a power transformation to attain constant variance and symmetric distribution. The symmetry of distribution, stability of variance and homogeneity of the transformed data and residuals were checked using diagnostic plots. This was followed by ANOVA analysis. The obtained results were transformed back to the original scale in order to be displayed graphically. The relationships between one dependent variable and the set of mutually intercorrelated explaining variables were analyzed using a multivariate regression with a reduction of dimensionality (orthogonal projections to latent structure, OPLS). To estimate the relationships between the dependent variables and the set of explaining variables independently of intercorrelations, the ordinary multiple regression was also applied. Similar to ANOVA testing, statistical analysis was carried out using OPLS analysis and multiple regression analysis was performed using the transformed data. Statistical software NCSS v. 12 from Number Cruncher Statistical Systems (Kaysville, Utah, United States), Statgraphics Centurion 18 v. 18. 1. 06 from Statgraphics Technologies Inc. (The Plains, Maryland, United States) and SIMCA v. 12. 0.0.0. from Umetrics (Umeå, Sweden) were used for the data analyses.

## Results

A total of 184 subjects were included into this study, 156 with suspicion of systemic infection and 28 healthy controls (HCs). The subjects excluded from the initial cohort of suspected infection were: newborns <34th week of gestational age (*n* = 11); newborns older than 72 h (*n* = 3); newborns with only a single time-point sampling of umbilical blood (*n* = 7); newborns who were sampled for insufficient quantity of blood (*n* = 2); and 1 newborn with abnormal EEG due to severe intrauterine hypoxia.

The remaining newborns (*n* = 132) were divided into 3 subgroups based on the criteria from *The Neonatal PCT Intervention Study* [(16), see Figure 1] which segregated as follows: unlikely infection = low-risk (R1; *n* = 81), possible infection = medium-risk (R2; *n* = 31), and probable infection = high-risk (R3; *n* = 20).

The “proven infection” subgroup (positive blood culture + total score ≥ 1) had to be omitted due to the absence of any positive blood culture results within the studied cohort.

There were no significant differences in birth weight, gender, gestational age, and APGAR score at 1 min among the subgroups. A significantly lower APGAR scores were observed at 5 min in the R2 group (*p* = 0.024) and at 10 min in the R2 & R3 subgroups (*p* < 0.001) compared to the HCs (R0). Higher frequency of caesarean sections was observed in groups R0 & R1 (low-risk) (Table 2).

**Table 2.** The selected clinical characteristics in each studied subgroup (R0 - healthy control, R1 – low-risk, R2 - medium-risk, R3 - high-risk)

Risk	R0	R1	R2	R3
Gestational age [week]	38.9 (38.2, 39.4)	38.6 (38.2, 39)	37.9 (37.1, 38.6)	39 (38.3, 39.7)
	Risk: F=1.5, p=0.208, *η*_p_^2^=0.0279			
Weight [g]	3230 (3030, 3440)	3190 (3070, 3310)	3110 (2920, 3310)	3460 (3220, 3690)
	Risk: F=1.3, p=0.274, *η*_p_^2^=0.024			
APGAR, min 1	8.9 (8.55, 9.21)	8.46 (8.23, 8.67)	8.36 (7.96, 8.71)	8.24 (7.71, 8.69)
	Risk: F=1.8, p=0.159, *η*_p_^2^=0.0316			
APGAR, min 5	9.34 (9.05, 9.61)	9.13 (8.95, 9.3)	8.64 (8.3, 8.95)	8.85 (8.44, 9.21)
	Risk: F=3.2, p=0.024, *η*_p_^2^=0.0551, R2<R0			
APGAR, min 10	9.73 (9.55, 9.9)	9.59 (9.47, 9.69)	9.16 (8.92, 9.37)	9.12 (8.81, 9.38)
	Risk: F=7, p<0.001, *η*_p_^2^=0.106, R2<R0, R3<R0			
Caesarean section	15/28 (53.57%)	30/81 (37.04%)	6/31 (19.35%)	4/20 (20.00%)
	p=0.021 (χ^2^ test), R2<R0, R3<R0			
Male gender	17/28 (60.71%)	41/81 (50.62%)	18/31 (58.06%)	13/20 (65.00%)
	p=0.593 (χ^2^ test)			

Continuous variables were evaluated by one way ANOVA followed by Bonferroni multiple comparisons (vs. control risk level R0) and factor variables were evaluated using χ^2^ tests. In addition to the overall χ^2^ tests, χ^2^ tests with Bonferroni correction were applied to compare individual risk levels with the control risk level R0. The symbol *η*_p_^2^ represents the effect size. Only significant multiple comparisons (p<0,05) are shown.

**Figure 2:**
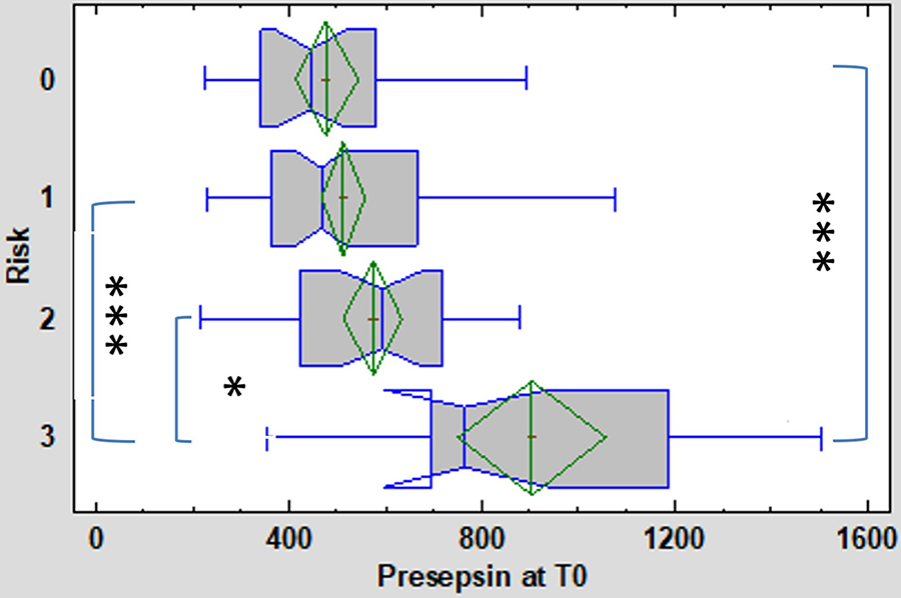
Presepsin (P-SEP) levels at T0 (onset of symptoms) in the 3 subgroups (low [1], medium [2], high-risk [3]) of enrolled neonates and healthy controls [0]. Kruskal-Wallis test followed by robust Dunn's multiple comparisons with Bonferroni correction was used to evaluate between group differences. Vertical lines symbolize medians, boxes indicate interquartile range, whiskers indicate the limits for data homogeneity, and crosses with rhombs symbolize means with SDs. ***p < 0.001, *p < 0.05.

At the time of first symptom onset (T0), the concentration of P-SEP was significantly higher in the high-risk group (median 766 ng/L, IQR 697–1189 ng/L) compared to the low-risk group (median 466 ng/L, IQR 363–663 ng/L; *p* = 0.0000025) and HCs (median 445 ng/L, IQR 339–582 ng/L; *p* = 0.0000013). In the medium-risk group, the P-SEP concentration (median 592 ng/L, IQR 423–720 ng/L) did not differ significantly from the low-risk group and HCs (Figure 2).

**Figure 3:**
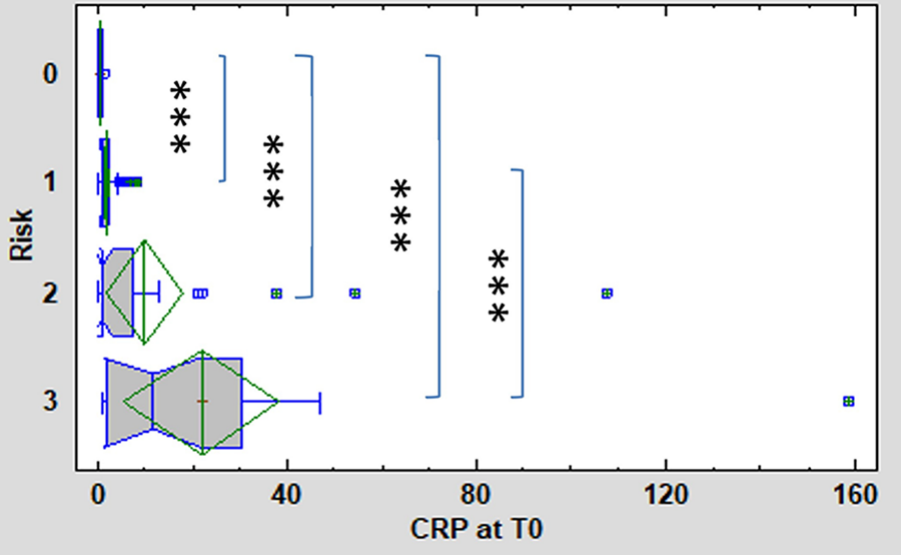
CRP levels at T0 (onset of symptoms) in the 3 subgroups (low [1], medium [2], high-risk [3]) of enrolled neonates and healthy controls [0]. Kruskal-Wallis test followed by robust Dunn's multiple comparisons with Bonferroni correction was used to evaluate between group differences. Vertical lines symbolize medians, boxes indicate interquartile range, whiskers indicate the limits for data homogeneity, and crosses with rhombs symbolize means with SDs. ***p < 0.001.

CRP concentration at symptoms onset (T0) differed between the high-risk group (median 11.4 mg/L, IQR 1.6–30.6 mg/L) compared to HCs (median 0.2 mg/L, IQR 0.1–0.6 mg/L), as well as between the medium-risk (median 1 mg/L, IQR 0.6–7.4 mg/L) and low-risk groups (median 0.6 mg/L, IQR 0.6–2.0 mg/L). A significant difference was also apparent between the low-risk and high-risk groups (Figure 3). The concentration of CRP did not differ significantly between the low-risk and medium-risk group and between the medium-risk and high-risk group.

Figure 4 displays the association of mean levels of P-SEP, IL-6, PCT and CRP with selected variables. A-D refer to the associations of each biomarker with the degree of risk of infection at the time of symptom onset (T0), E-H illustrate the association of each biomarker with sampling timepoints, CH-K depict the resulting interaction between both variables.

**Figure 4:**
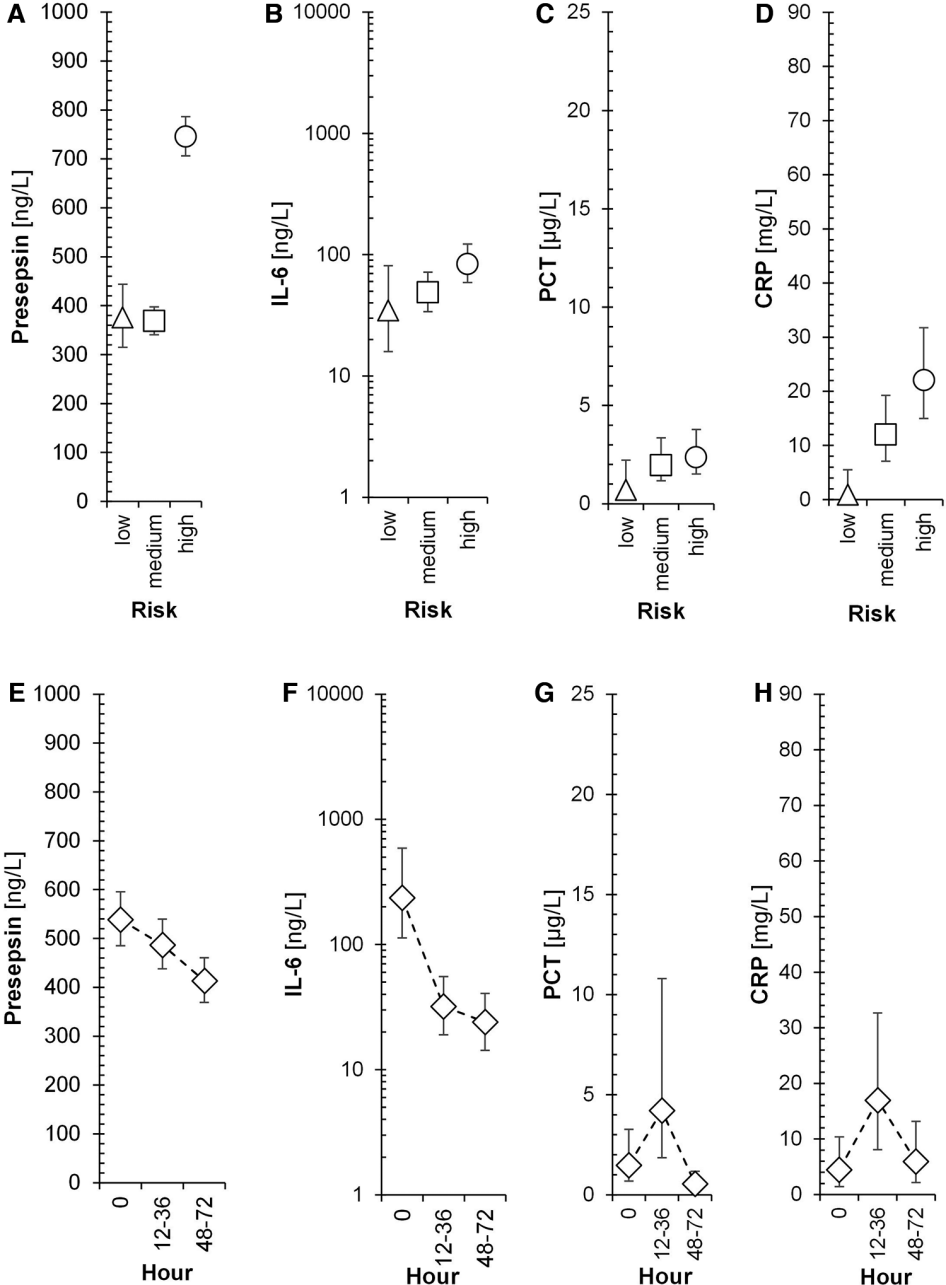
Time-dependent dynamics of presepsin and other biomarkers in the 3 subgroups of neonates. Triangles, squares, and circles with error bars symbolize re-transformed means with their 95% confidence intervals after Bonferroni correction for low-risk, medium-risk and high-risk group, respectively as evaluated by ANOVA model consisting of between subject factor Risk of infection, within-subject factor Hour, and Subject factor (explaining inter-individual variance) followed by Bonferroni multiple comparisons. Presepsin-Risk: *F* = 113.9, *p* < 0.001, *η*_p_^2^ = 0.37; Hour: *F* = 5.5, *p* = 0.013, *η*_p_^2 ^= 0.0276; Risk × Hour: *F* = 1.6, *p* = 0.209, *η*_p_^2 ^= 0.0165; Subj(Risk): *F* = 8.9, *p* < 0.001, *η*_p_^2 ^= 0.186; IL-6-Risk: *F* = 2.8, *p* = 0.088, *η*_p_^2 ^= 0.0362; Hour: *F* = 13.7, *p* < 0.001, *η*_p_^2 ^= 0.157; Risk × Hour: *F* = 1.9, *p* = 0.147, *η*_p_^2 ^= 0.0498; Subj(Risk): *F* = 3, *p* = 0.018, *η*_p_^2^ = 0.171; PCT-Risk: *F* = 1.7, *p* = 0.214, *η*_p_^2 ^= 0.0388; Hour: *F* = 5.7, *p* = 0.011, *η*_p_^2 ^= 0.121; Risk × Hour: *F* = 0.4, *p* = 0.836, *η*_p_^2 ^= 0.017; Subj(Risk): *F* = 3.9, *p* = 0.005, *η*_p_^2 ^= 0.321; CRP-Risk: *F* = 7.9, *p* = 0.003, *η*_p_^2 ^= 0.169; Hour: *F* = 2.7, *p* = 0.091, *η*_p_^2 ^= 0.0653; Risk × Hour: *F* = 0.5, *p* = 0.733, *η*_p_^2 ^= 0.0254; Subj(Risk): *F* = 2.4, *p* = 0.05, *η*_p_^2 ^= 0.233; *F*, *p*, and *η*_p_^2^ represent *F*-statistic, *p*-value, and effect size, respectively; *η*_p_^2 ^= 0.01, 0.06, and >0.14 are small, medium, and large effect size.

**Figure 4:**
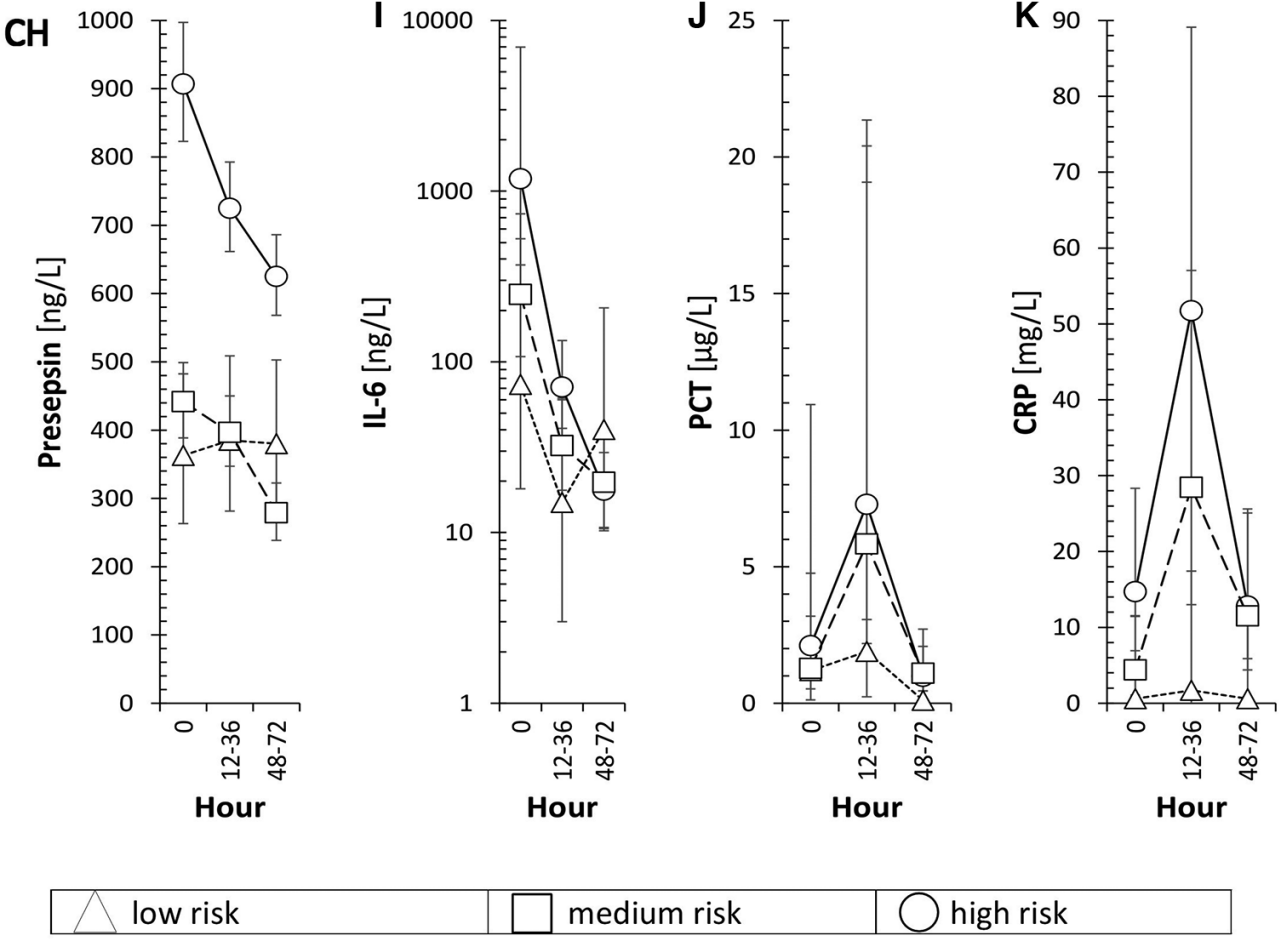
Risk, hour and both variables of individual inflammatory markers is in the text of Figure 4.

The association of mean levels of P-SEP, IL-6, PCT and CRP with the risk of infection at T0 was strong for P-SEP (Figure 4A, *p* < 0.001) and CRP (Figure 4D, *p* = 0.003) and weak for PCT (Figure 4C, *p* = 0.214) and IL-6 (Figure 4B, *p* = 0.088). All evaluated inflammatory parameters, except of CRP (Figure 4H, *p* = 0.091), demonstrated a strong dependency on the time of sampling (Figures 4E–H). The increase of PCT at 12–36 h from T0 did not reach expected statistical significance; however, the decline in its concentration at 48–72 h was significant (*p* < 0.05). The concentrations of P-SEP and IL-6 did not increase past T0 values at subsequent samples but, unlike PCT and CRP, their levels, at post-initial samples showed significant decreases (Figure 4E, *p* = 0.013; Figure 4F *p* < 0.001).

**Figure 5:**
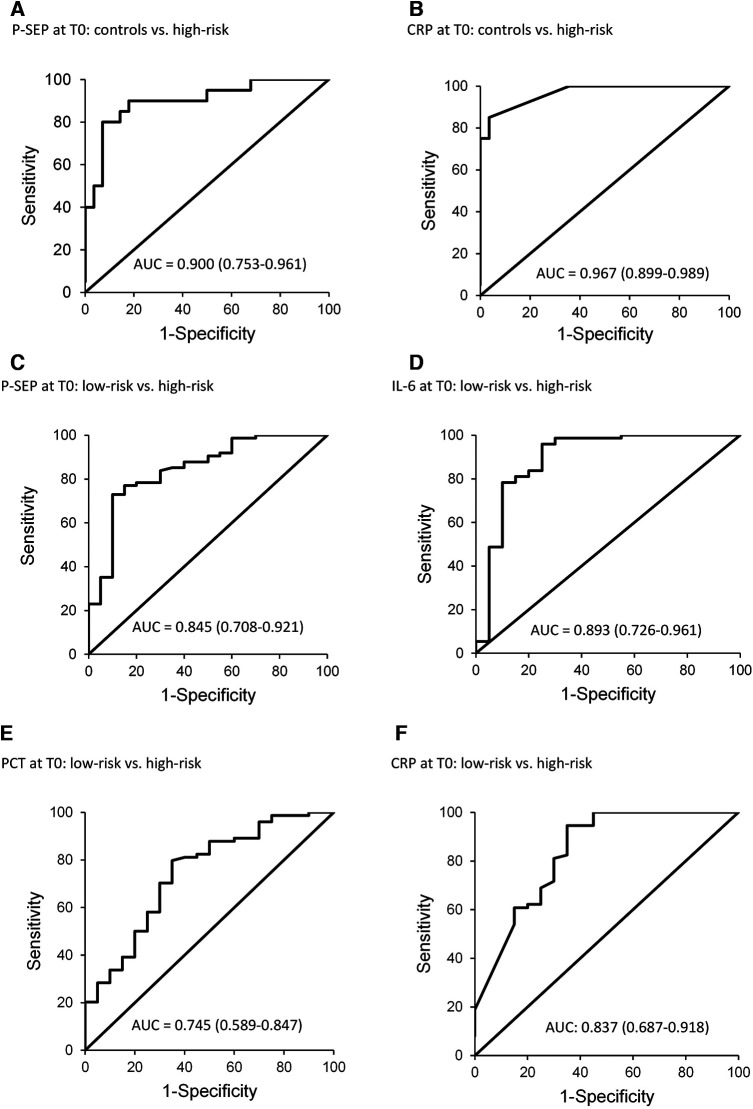
ROC curves for P-SEP, CRP at T0 (onset of symptoms) in healthy controls, and in the group low-risk extra PCT and IL-6 at T0 (onset of symptoms) in the group low-risk.

The sensitivity and specificity of P-SEP across the studied subgroups at T0 were evaluated, compared to the low-risk group and HCs (Table 3; Figure 5).

**Table 3.** Diagnostic criteria of P-SEP at T0 using HCs/low-risk group as reference. PPV: positive predictive value, NPV: negative predictive value, POS LR: positive likelihood ratio. NEG LR: negative likelihood ratio. Confidence intervals are in parentheses.

Group	Max. Youden index	Sensitivity	Specificity	PPV	NPV	POS LR	NEG LR
**Reference Group: Healthy neonates**							
High risk	0.729	0.800	0.929	0.889	0.867	11.2 (2.9, 43.3)	0.215 (0.089, 0.521)
**Reference Group: low-risk group**							
High risk	0.616	0.900	0.716	0.462	0.964	3.171 (2.147, 4.686)	0.14 (0.037, 0.524)

Figure 5 depicts the diagnostic power of P-SEP, i.e., its ability to discriminate newborns at high-risk from healthy and low-risk newborns (Figures 5A,C). The observed differences between HCs [AUC = 0.9 (0.753–0.961)] and low-risk groups [AUC = 0.845 (0.708–0.921)] were not significant (see Figure 2).

Further, Figures 5D–F shows the diagnostic power of other inflammatory parameters compared to the low-risk group, due to the higher number of subjects in the group (*n* = 81) and the evaluation of all inflammatory markers compared to the control group, where only P-SEP and CRP levels were evaluated.

Only IL-6 [AUC = 0.893 (0.726–0961)] (Figure 5D) demonstrated better differentiation between the groups (low vs. high-risk). The area under the curve for CRP [AUC = 0.837 (0.687–0.918)] showed similar differentiation performance as P-SEP (Figure 5F). PCT exhibited the worst differentiation potential [AUC of 0.745 (0.589–0.847)].

**Figure 6:**
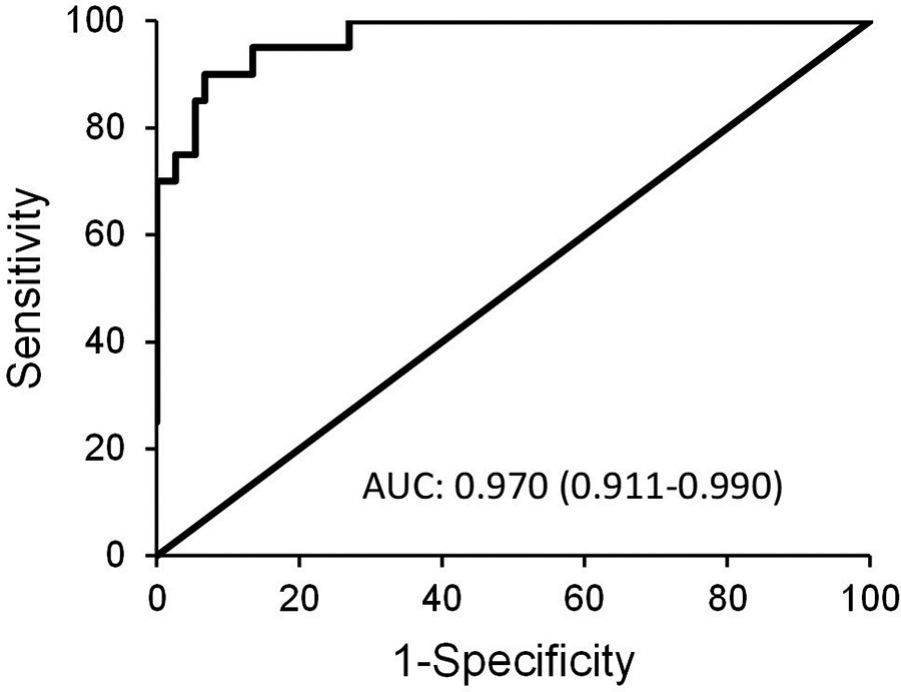
ROC curve depicting the diagnostic power of combined use of P-SEP with IL-6 and CRP in between-group comparison of low-risk vs. high-risk group.

The highest diagnostic efficacy was achieved when P-SEP was used in combination with IL-6 and CRP. This is illustrated in Figure 6, which compares the high-risk group to the low-risk group [AUC = 0.97 (0.911–0.99)], attaining a 90% sensitivity (76.9–103.1), 93.2% specificity (87.5–99), PPV 78.3% (61.4–95.1), negative predictive value (NPV) 97.2% (93.3–101), LR− 0.107 (0.029–0.4) and LR+ 13.32 (6.643–31.443).

A cut-off value of P-SEP at 636 ng/L (AUC 0.845; 0.708–0.921) had low PPV (46.2%) in the low-risk group comparison. Also, the value was close to the literature-published values of healthy neonates (603.5 ng/L, IQR 466.5–791 ng/L) and preterm neonates (620 ng/L, IQR 503–864 ng/L) (17). P-SEP demonstrated a better PPV when the reference group was healthy neonates. In this case, the cut-off value was set to 695 ng/L (AUC 0.9; 0.753–0.961) with sensitivity of 80% (62.5–97.5), specificity 92.9% (83.3–102.4), PPV 88.9% (74.4–103.4), NPV 86.7% (74.5–98.8), LR− 0.215 (0.089–0.521) and LR+ 11.2 (2.894–43.349) (Table 3).

Lastly, a significant difference in P-SEP concentrations between the T0 values and the samples obtained in 48–72 h interval was observed in the low-risk group. However, due to the limited number of sequentially obtained samples no further correlation was possible.

## Discussion

Sepsis is one of the most common causes of death in pediatric population, especially in the neonates. Early initiation of treatment reduces mortality and morbidity, thus a prompt diagnosis is essential.

The existing consensus on pediatric sepsis definitions (sepsis-3) (6, 7) have, however, limited accuracy in term neonates and are not appropriate for preterm infants. This is mainly because the organ dysfunction, the key diagnostic criterion, is rarely considered in neonatal literature, and it remains unclear how to screen neonates most accurately for organ dysfunction (4).

The gold standard method for confirming sepsis in newborns with risk factors, clinical suspicion and abnormal test results is the identification of a pathogenic organism from otherwise sterile site (blood or cerebrospinal fluid) (14). However, blood cultures lack sensitivity due to the specific characteristics of the neonatal population (18) and are positive in less than 1% of EOS cases (19, 20). The ability to detect bacteremia in neonates with EOS is significantly reduced by administering intrapartum antibiotic prophylaxis to at risk mothers, due to transplacental transfer of antibiotics to the fetus (9–11). Also, the recommended blood volume for culture in newborns is 1–3 ml, which is often impracticable to obtain in the first hours/days of life, especially when other tests have to be prioritized. In practice, the majority of samples are less than 0.5 ml (21). In addition, over half of septic newborns (68%) have low-colony count bacteremia [≤10 colony forming units (CFU)/ml], and as many as 60% of cultures will be interpreted as falsely negative with 0.5 ml sample volumes (22). In fact, even multiple blood cultures do not increase the yield of the methodology (23). Last but not least, even in the most efficient operations the culture results are typically not available sooner than in 24 h, which is an unacceptably long delay to treatment initiation in a septic infant.

For these reasons, biomarkers of sepsis are commonly used to aid early sepsis diagnosis together with the clinical signs.

In this work, the sepsis criteria of the NeoPInS study (16) were used to stratify the newborns. Correlating with previous experience (24), no positive blood culture samples were captured, as more than 1,000 newborns would have to be collected to capture 1 positive blood culture sample (19, 20). Therefore, newborns with risk factors, clinical signs and abnormal test results were classed as those with EOS, regardless of the negativity of blood culture.

Previously published data supported the use of P-SEP in the diagnosis of EOS, suggesting its similar or higher diagnostic accuracy compared to other markers of inflammation (25, 26). However, conflicting results were reported on whether P-SEP alone may be sufficient in the diagnosis of EOS (27) or whether it may be used to better advantage in combination with other inflammatory markers (28–31). According to our observations, P-SEP alone exhibited, an undisputable discriminatory potential between high and low-risk/healthy subjects, however a higher diagnostic efficacy was achieved by combining P-SEP with IL-6 and CRP (see Table 4). Comparing the individual performance of P-SEP, CRP, PCT and IL-6, IL-6 demonstrated the best discriminatory power and PCT the worst. When P-SEP was used together with IL-6 and CRP, their combined NPV was the highest**.** In addition, the concentration of P-SEP in the low-risk and medium-risk groups did not differ significantly from that of HCs. Therefore, by theoretical extension, which would include confirmed cases of sepsis, P-SEP can be used as a negative predictive marker of EOS, especially in neonates at low to moderate risk of infection.

**Table 4.** Sensitivity, specificity, NPV, PPV, POS LR and NEG LR for P-SEP alone and P-SEP in combination with other inflammatory markers (CRP, IL-6). NPV: negative predictive value, PPV: positive predictive value, POS LR: positive likelihood ratio, NEG LR: negative likelihood ratio (confidence intervals are in parentheses).

Parameter	P-SEP	P-SEP + CRP	P-SEP + IL-6	P-SEP + CRP + IL-6
Sensitivity	0.9 (0.769, 1.031)	0.85 (0.694, 1.006)	1.000 (1.000, 1.000)	0.900 (0.769, 1.031)
Specificity	0.716 (0.613, 0.819)	0.851 (0.770, 0.932)	0.878 (0.804, 0.953)	0.932 (0.875, 0.990)
PPV	0.462 (0.305, 0.618)	0.607 (0.426, 0.788)	0.679 (0.506, 0.852)	0.783 (0.614, 0.951)
NPV	0.964 (0.914, 1.013)	0.955 (0.904, 1.005)	1 (1, 1)	0.972 (0.933, 1.010)
POS LR	3.171 (2.147, 4.686)	5.718 (3.216, 10.167)	8.222 (4.457, 15.168)	13.320 (5.643, 31.443)
NEG LR	0.14 (0.037, 0.524)	0.176 (0.062, 0.502)	N/A	0.107 (0.029, 0.400)
Overal accuracy	0.755 (0.668, 0.842)	0.851 (0.779, 0.923)	0.903 (0.843, 0.963)	0.926 (0.872, 0.979)

Relationships between low-risk vs. high-risk group (logarithm of the likelihood ratio, LLR) and predictors as evaluated by multivariate regression, with a reduction of dimensionality know as orthogonal projections to latent structure (OPLS). The statistical software SIMCA-P v.12.0 from Umetrics AB (Umeå, Sweden) was used for OPLS analysis. Only significant multiple comparisons (p<0,01) are shown.

Thus, the introduction of P-SEP into clinical practice can help prevent false positive diagnoses of sepsis, thereby limiting neonates' exposure to antimicrobial drugs, their adverse events and invasive procedures (32).

In this study, P-SEP cut-off value at 695 ng/L has 80% sensitivity and 92.9% specificity. The positive and negative predictive values were 88.9% and 86.7%, respectively. Other studies (33, 34) suggested a higher P-SEP cut-off was associated with higher sensitivity and NPV.

Our study had several limitations. Firstly, the number of enrolled neonates is limited and the quantitative heterogeneity of the studied subgroups may influence the precision of statistical analysis. Secondly, the inability to assess a group of newborns with confirmed sepsis due to the absence of positive blood culture samples within the cohort represents a risk of an overestimation of P-SEP accuracy and may affect the P-SEP cut-off point. Thirdly, our results may be influenced by other factors which could affect P-SEP concentrations. While previous studies suggested that non-infectious sources of inflammation should not markedly alter P-SEP levels (35–37), other factors, such as the use of chemotherapeutics in the infants or their mothers, may do so. Finally, this study did not include very preterm and extremely preterm newborns. Based on our data, we suggest that the performance of P-SEP in these groups should be studied in future.

## Conclusion

Our study showed that P-SEP may be a useful biomarker in prompt detection of early-onset sepsis in late preterm and term newborns, however it's not individually superior to IL-6. When P-SEP was used in combination with IL-6 and CRP, the best negative predictive power was achieved, especially in newborns at low to medium risk of infection. We suggest a P-SEP cut-off point at 695 ng/L. On the other hand, PCT was the least efficient amongst the studied markers, but further studies are needed to rule out any confounders.
